# Preoperative evaluation of microvascular invasion in hepatocellular carcinoma with a radiological feature-based nomogram: a bi-centre study

**DOI:** 10.1186/s12880-024-01206-7

**Published:** 2024-01-27

**Authors:** Yuhui Deng, Dawei Yang, Xianzheng Tan, Hui Xu, Lixue Xu, Ahong Ren, Peng Liu, Zhenghan Yang

**Affiliations:** 1 Department of Radiology, Beijing Friendship Hospital, Capital Medical University, Yongan Road 95, West District, Beijing, 100050 China; 2 Medical Imaging Division, Heilongjiang Provincial Hospital, Harbin Institute of Technology, Zhongshan Road 82, Xiangfang District, Harbin, 150036 China; 3 Department of Radiology, Hunan Provincial People’s Hospital, the First Affiliated Hospital of Hunan Normal University, Changsha, 410005 China

**Keywords:** Hepatocellular carcinoma, Microvascular invasion, CT, MRI, Nomogram

## Abstract

**Purpose:**

To develop a nomogram for preoperative assessment of microvascular invasion (MVI) in hepatocellular carcinoma (HCC) based on the radiological features of enhanced CT and to verify two imaging techniques (CT and MRI) in an external centre.

**Method:**

A total of 346 patients were retrospectively included (training, *n* = 185, CT images; external testing 1, *n* = 90, CT images; external testing 2, *n* = 71, MRI images), including 229 MVI-negative patients and 117 MVI-positive patients. The radiological features and clinical information of enhanced CT images were analysed, and the independent variables associated with MVI in HCC were determined by logistic regression analysis. Then, a nomogram prediction model was constructed. External validation was performed on CT (*n* = 90) and MRI (*n* = 71) images from another centre.

**Results:**

Among the 23 radiological and clinical features, size, arterial peritumoral enhancement (APE), tumour margin and alpha-fetoprotein (AFP) were independent influencing factors for MVI in HCC. The nomogram integrating these risk factors had a good predictive effect, with AUC, specificity and sensitivity values of 0.834 (95% CI: 0.774–0.895), 75.0% and 83.5%, respectively. The AUC values of external verification based on CT and MRI image data were 0.794 (95% CI: 0.700–0.888) and 0.883 (95% CI: 0.807–0.959), respectively. No statistical difference in AUC values among training set and testing sets was found.

**Conclusion:**

The proposed nomogram prediction model for MVI in HCC has high accuracy, can be used with different imaging techniques, and has good clinical applicability.

## Background

As a most common primary liver malignancy, hepatocellular carcinoma (HCC) is among the top three causes of tumour-linked death in the world [1, 2]. Liver transplantation and surgical excision are currently the best treatment options and have been continuously furthered recently. However, because of the high recurrence rate, the early and long-term prognoses of HCC are still not ideal even after treatment [3]. Microvascular invasion (MVI) has been proved to be an important factor in the high recurrence rate of patients with HCC after resection or transplantation [4, 5]. Wide-margin surgery for MVI of HCC has been shown to reduce postoperative recurrence [6, 7]. However, MVI is a postoperative pathological diagnosis, and a noninvasive, high-precision tool is needed to assess the presence of MVI in HCC to assist in making appropriate preoperative treatment decisions.

With the characteristics of noninvasive assessment of blood supply, water molecular diffusion restriction, hepatic function and more clearly showing morphologic changes, multiple magnetic resonance imaging (MRI) techniques have been utilized to noninvasively evaluate MVI status in HCC. A meta-analysis [8] revealed that the apparent diffusion coefficient (ADC) value alone had medium accuracy to predict MVI in HCC with a pooled sensitivity, specificity and area under the receiver operating characteristic curve (AUROC) of 0.73, 0.70, and 0.78, respectively. A diagnostic accuracy of imaging features in gadoxetic acid-enhanced MRI was also assessed in another meta-analysis [9], which showed a comparable accuracy to the ADC value, with AUROCs in the range of 0.74 to 0.76. However, the use of only imaging features without the consideration of clinical indices might reduce the prediction accuracy. Recently, machine learning techniques such as radiomics or deep learning models have been used to improve the efficacy of predictive methods [10–13], which have shown better diagnostic accuracy. However, considering the requirement of specialized software, weak robustness, and unsatisfactory generalization performance interhospitally, machine learning currently remains too idealized to be extensively used, especially in clinics without the appropriate conditions to apply this novelty technique.

In practise, dynamic contrast-enhanced MRI (DCE-MRI) and contrast-enhanced CT (CE-CT) remain the most valuable techniques in the detection and diagnosis of HCC in cirrhosis. Dynamic contrast enhancement technique can capture the enhancement characteristics of tumors at different periods, which is conducive to the diagnosis and differential diagnosis of liver tumors. In addition, dynamic techniques such as perfusion CT and MRI can quantify perfusion of hepatocellular carcinoma, which is currently an important means to evaluate the effectiveness of sorafenib therapy [14–16]. All of these can be used as a supplement to conventional imaging to guide diagnosis and treatment. Meanwhile, the recognition of imaging features on DCE-MRI or CE-CT is not challenging for radiologists and experienced surgeons. Previous studies have revealed that imaging features such as tumour margins, tumour size, tumour capsule, intratumoural artery, and arterial peritumoral enhancement were correlated with the status of MVI in HCC, [17–21] and these features could be well captured on DCE-MRI or CE-CT. For example, Ling et al. found rim enhancement in the arterial phase and peritumoral hypointensity in the hepatobiliary phase were independent risk factors for microvascular invasion in patients with HCC [18]. Matteo et al. found tumor dimension, nonsmooth tumor margins, peritumoral enhancement, and TTPVI, had high accuracy in the prediction of MVI in HCC [19]. And Wei et al. found capsular invasion, margins and serum AFP level were associated with MVI in HCC [20]. However, few studies among them have considered these imaging features in combination with clinical indices to build a nomogram model for the prediction of MVI and verify it in different medical centres.

Therefore, the study proposed to develop a clinically practical nomogram model based on imaging features from CE-CT and clinical indices to predict MVI in HCC and to verify its generalization on external data, including both MRI and CT data.

## Methods

The patients were included from two centres, including Hunan Provincial People's Hospital, the First Affiliated Hospital of Hunan Normal University (Centre I) and Beijing Friendship Hospital, Capital Medical University Affiliated Hospital (Centre II). This retrospective study was authorized by agency review board, waiving the requirement for informed consent.

### Patients

**Fig. 1:**
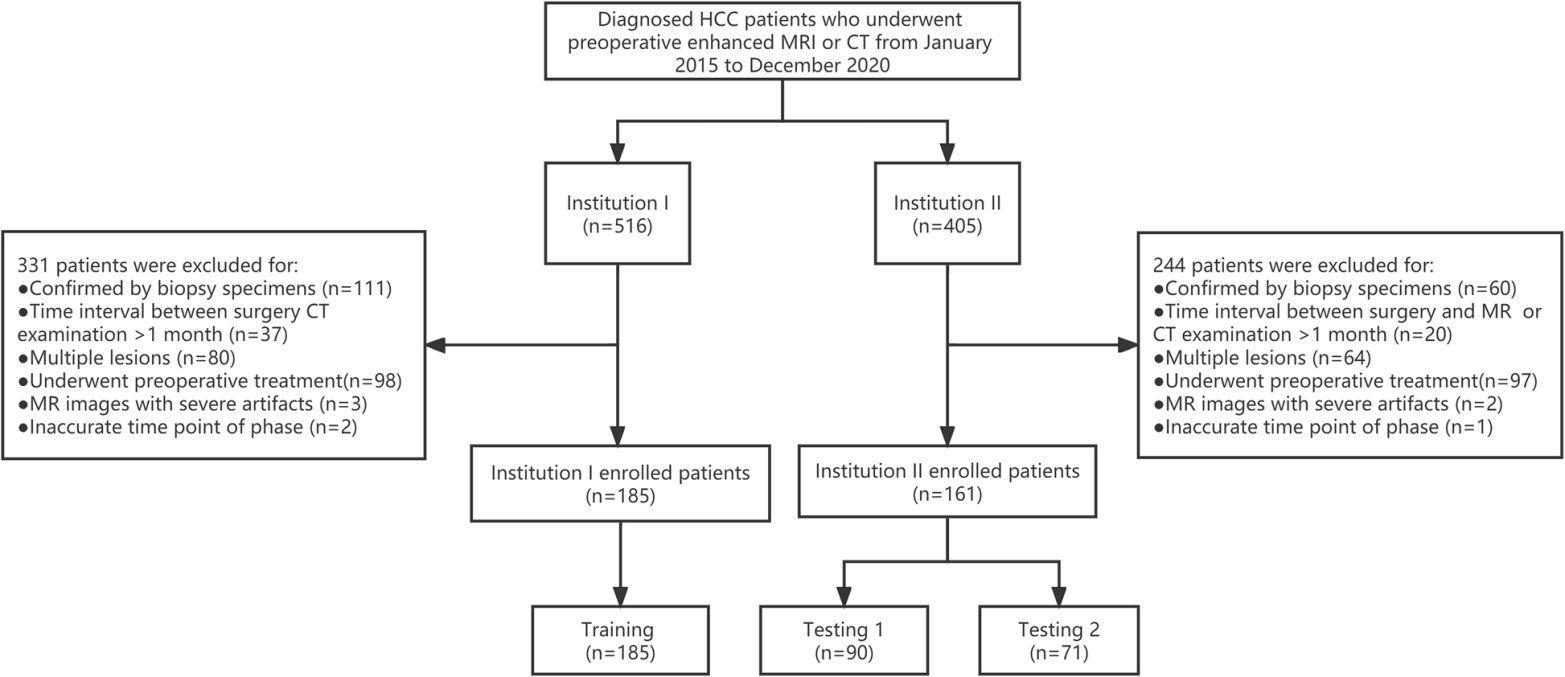
Flow Chart. HCC hepatocellular carcinoma, MRI Magnetic Resonance Imaging, CT computed tomography

HCC patients who underwent hepatectomy or liver transplantation at two hospital centres and were diagnosed between January 2015 and December 2020 were considered. The inclusion criteria as follows: (1) dynamic enhanced MRI (DCE-MRI) and/or enhanced CT (CE-CT) images of the liver were obtained, including at least pre-enhanced, arterial phase, portal phase, and equilibrium phase images; (2) there was no percutaneous ethanol injection, transcatheter arterial chemoembolization or radiofrequency ablation; and (3) the pathologic status of MVI in HCC was obtained from surgical resection specimens. The exclusion criteria as follows: (1) the time point of the reinforcement stage was not accurate; (2) the interval time between CT or MRI examination and surgery was more than one month; (3) there were significant artefacts affecting the HCC observation; and (4) multiple lesions were present. Details are provided in the flowchart in Fig. 1.

Overall, 346 consecutive patients (185 patients from Centre I and 161 patients from Centre II) were enrolled, and the CT imaging data from Centre I were set as a training cohort. The CT or MRI data of Centre II were used as the external validation cohort, in which the CT imaging data were used as trial group Testing 1 and the MRI imaging data were used as Testing 2.

### Clinical characteristics

Clinical information and preoperative laboratory tests were obtained from our case database, including age, tumour size, aetiology of liver disease, sex, alkaline phosphatase (ALP), serum alanine aminotransferase (ALT), international normalized ratio (INR), aspartate aminotransaminase (AST), glutamyl-transpeptidase (GGT), prothrombin time (PT), serum albumin (ALB), platelet count (PLT), serum total bilirubin (TB), Child‒Pugh grade, background liver, and alpha-fetoprotein (AFP).

### Image acquisition

CE-CT scanning of the liver was performed using multiple multislice spiral CT scanners. Scan from the roof of the diaphragm to the iliac ridge. It includes plain scan, arterial stage (fluoroscopic trigger, 28 ~ 35 s), portal vein stage (70-80 s) and equilibrium stage (180 s). All patients received a non-ionic iodide contrast (300 mg of iodide per ml) at a dose of 1.5 ml (450 mg of iodide) per kg of body weight with a flow rate of 3.0–4.0 ml/s. Detailed imaging parameters of CT are shown in Table 1.

**Table 1:** Detailed imaging parameters of CE-CT

	Philips ICT	GE Revolution	GE Discovery CT750HD
No. of channels	256	256	256
Tube voltage (kV)	120	120	120
Tube current (mA)	420	450	450
Helical pitch	0.991	0.992	0.984
Acquisition time (s)	2–6	2–6	2–6
Section thickness (mm)	1–5	1.25–5	1–5
Intersection gap	0	0	0
Reconstruction kernel	soft tissue	standard	standard

All MRI examinations are performed on a 3.0 T scanner using 16–64 channel phased array coils. Scanning sequences include: fast spin echo T2-weighted imaging, diffusion-weighted sequences (B-values: 0 s/mm2 and 800 s/mm2), (3D) gradient echo T1-weighted sequence enhanced anterior imaging, Arterial phase (AP) of 15 to 25 s, Portal venous phase (50 s), PVP), 180 s Equilibrium phase (EP). The standard dose (0.1 mmol/kg) of Gadopentetic acid (Gd-DTPA) was injected at a flow rate of 2.0 ml/s. Detailed imaging parameters of MRI are shown in Table 2.

**Table 2:** Detailed imaging parameters of MRI

Sequence	MRI unit	TR (ms)	TE (ms)	Flip angle (°)	Matrix	FOV (mm2)
T2-weighted imaging	3.0 T Siemens Prisma 3.0 T GE Healthcare GE 750w 3.0 T Philips Ingenia	2160 6315 4918	100 85 106	160 150 160	320 × 288 288 × 244 288 × 224	433 × 433 360 × 280 285 × 380
Diffusion-weighted imaging	3.0 T Siemens Prisma 3.0 T GE Healthcare GE 750w 3.0 T Philips Ingenia	5600 3000 5100	Minimum Minimum 55	90 90 90	100 × 76 128 × 128 128 × 128	380 × 289 360 × 380 285 × 380
Dynamic T1-weighted imaging	3.0 T Siemens Prisma 3.0 T GE Healthcare GE 750w 3.0 T Philips Ingenia	3.95 4.1 3.47	Minimum Minimum 1.36	9 15 10	352 × 256 288 × 172 320 × 216	400 × 296 380 × 300 308 × 380

### Pathological diagnosis of MVI in HCC

Surgical specimens were histopathologically examined by two experienced pathologists, to whom the patient's imaging findings and clinical history were not visible. MVI in HCC was ruled as the microscopic discovery of tumour thrombus in small peritumoral vessels, which may be hepatic veins, portal veins, or large capsular vessels aligned with the surrounding liver tissue. If there were any differences, they were resolved through consultation.

### Image analysis

All images were evaluated by two radiologists (with 10 + years of experience in abdominal imaging) with knowledge of HCC but not knowledge of the pathology associated with MVI. In the event of a disagreement over the above procedure, a third radiologist (with 15 + years of experience in abdominal imaging) helped to reach an agreement. At the same time, the Kappa value between observers was calculated, and a Kappa value greater than 0.75 was considered to indicate good repeatability.

Each patient was evaluated for the following imaging features: (1) radiological capsule, which was defined as the high-density or signal ring around the tumour in the portal phase or equilibrium phase and was classified as complete, incomplete, or absent; (2) tumour margin, which was divided into smooth and non-smooth (smooth tumour margins were defined as nodular tumours with smooth profiles in all imaging planes and vice versa); (3) arterial peritumoral enhancement (APE), which was defined as detectable enhancement near the tumour boundary in the AP, which then became equidecayed in the equilibrium phase; (4) hypoattenuating halo, which was defined as a partially or completely low-density or signal ring surrounding the tumour in the portal phase; (5) intratumoural arteries, which were defined as the internal artery presented in the arterial phase; and (6) arterial rim enhancement (ARE), which was defined on arterial phase images as irregularly rim-like peripheral hyperenhancement and a hypoenhancing area in the centre.

### Establishment and evaluation of the nomogram prediction model

First, independent predictors of MVI in HCC were identified by multiple logistic regression in clinical and radiological features. Then, a nomogram prediction model was established by thees significant factors. The degree of generalization of the model was evaluated by external validation using external centre data based on different imaging techniques. Harrell's C-index was used to evaluate the discriminability of the nomogram [22]. The nomogram diagnostic performance of the training and testing cohorts was analysed by calibration curves [23]. The consistency between the prediction of MVI and the actual MVI on the calibration curve was evaluated using the Hosmer‒Lemeshow test [24]. The clinical usefulness of the nomogram was determined by decision curve analysis [25].

### Statistical analysis

Statistical analysis was performed using R software (version 3.6.1, Boston, MA, USA) and SPSS (version 26, Chicago, IL, USA). The mean ± standard deviation are used for continuous variable. The number (percentage) are used for categorical variables. Pearson's chi-square test was utilized to evaluate categorical variables, and the Mann‒Whitney U test or Student's t test was utilized to evaluate continuous variables. Multivariate logistic regression analysis, nomogram prediction model construction, calibration curve, external validation, ROC curve, and decision curve ananlysis were carried out using various packages in R language. The AUC value, accuracy, sensitivity and specificity were recorded. A two-tailed *p* value lower than 0.05 was defined statistically significant.

## Results

### Demographic data

The demographic data of 346 patients from the two centres are displayed in Table 3. The mean age was 55.85 years (26–82 years). There were 280 males (47.4%) and 66 females (36.9%). There were 229 MVI-negative cases (66.2%) and 117 MVI-positive cases (33.8%). No significant differences were found in sex, age or MVI status among the three datasets (*p* = 0.088, 0.328, 0.940).

**Table 3:** Demographic data in the training, testing 1 and testing 2

Variable	Total (*n* = 346)	Training (*n* = 185)	Testing 1 (*n* = 90)	Testing 2 (*n* = 71)	*p*-Value
Age (years, mean ± SD)	55.85 ± 10.86	54.73 ± 11.21	56.53 ± 11.08	57.90 ± 9.32	0.088
Sex (n, %)					
Male	280(80.9)	155(83.8)	69(76.7)	56(78.9)	0.328
Female	66(19.1)	30(16.2)	21(23.3)	15(21.1)	
MVI status (n, %)					
Positive	117(33.8)	64(34.6)	30(33.3)	23(32.4)	0.940
Negative	229(66.2)	121(65.4)	60(66.7)	48(67.6)	

*MVI* microvascular invasion, *SD* standard deviation

### Clinical and imaging characteristics

Sixty-four of 185 lesions in the training dataset, 30 of 90 lesions in the testing 1 dataset and 23 of 71 lesions in the testing 2 dataset were confirmed to be positive for MVI by histopathology, which were based on CE-CT, CE-CT and DCE-MRI images, respectively. The general characteristics of the included cohorts are summarized in Table 4. Univariate analysis revealed that the clinical factors tumour size and serum AFP level and the imaging features tumour margins and APE were significantly correlated with MVI (*p* < 0.05) in all three datasets. The imaging features additional APE in the training dataset and site and intratumoural arteries in the testing 2 dataset were significantly correlated with MVI (*p* < 0.05). Tumour size (OR: 1.061; 95% CI: 1.020–1.104;* p* = 0.003), AFP level (OR: 2.008; 95% CI: 1.144–23.526; *p* = 0.015), tumour margin (OR: 2.645; 95% CI: 0.1211–5.775;* p* = 0.015), and APE (OR: 2.556; 95% CI: 1.085–6.021; *p* = 0.032) were independent predictors of MVI in multivariate analysis. From the perspective of clinical factors, the greater the AFP value is, the larger the tumour, and the greater the possibility of MVI. For the image features, MVI is more likely to appear when the tumour margins are not smooth and the tumour has APE. The Kappa values between observers were 0.832 ~ 0.876 for radiological features.

**Table 4:** Clinical and radiological characteristics of all patients of Mvi ( +) And Mvi (-) groups in the training, testing 1 and testing 2

	Training Cohort (*n* = 185)		*P*-Value	Testing Cohort 1 (*n* = 90)		*P*-Value	Testing Cohort 2 (*n* = 71)		*P*-Value	*P*-Value*
	MVI (-) = 121	MVI ( +) = 64		MVI (-) = 60	MVI ( +) = 30		MVI (-) = 48	MVI ( +) = 23		
Clinical information										
Age,years	54.40 ± 11.30	55.34 ± 11.09	0.589	56.03 ± 12.72	56.78 ± 10.27	0.764	57.88 ± 10.49	57.96 ± 6.42	0.973	
Gender			0.794			0.113			0.478	
Male	102(84.3)	53(82.8)		49(81.7)	20(66.7)		39(75)	17(69.6)		
Female	19(15.7)	11(17.2)		11(18.3)	10(33.3)		9(25)	6(36.4)		
Etiolgy of liver disease			0.224			0.170			0.749	
HBV	106(87.6)	51(79.7)		47(78.4)	25(41.7)		34(70.8)	18(78.3)		
HCV	3(2.5)	1(1.5)		2(3.3)	3(5)		3(6.3)	3(13)		
other	12(9.9)	12(18.8)		11(18.3)	2(3.3)		11(22.9)	2(8.7)		
ALT			0.867			0.454			0.08	
< 40	76(62.8)	41(64.1)		31(51.7)	18(60)		25(52.1)	17(73.9)		
> 40	45(37.2)	23(35.9)		29(48.3)	12(40)		23(47.9)	6(26.1)		
AST			0.209			1			0.678	
< 35	74(61.2)	33(51.6)		30(50)	15(50)		29(60.4)	15(65.2)		
> 35	47(38.8)	31(48.4)		30(50)	15(50)		19(39.6)	8(34.8)		
ALP			0.372			0.209			0.335	
< 135	106(86.6)	53(82.8)		49(81.7)	21(70)		45(93.8)	20(87)		
> 135	15(13.4)	11(17.2)		11(18.3)	9(30)		3(6.3)	3(13)		
GGT			0.624			0.371			0.265	
< 60	80(66.1)	40(62.5)		32(53.3)	13(43.3)		29(60.4)	17(73.9)		
> 60	41(33.9)	24(37.5)		28(46.7)	17(56.7)		19(39.6)	6(26.1)		
ALB			0.996			0.134			0.885	
< 40	70(57.9)	37(57.8)		36(60)	13(43.3)		28(58.3)	13(56.5)		
> 40	51(42.1)	27(42.2)		24(40)	17(56.7)		20(41.7)	10(43.5)		
PT			0.696			0.858				
< 13.5	110(90.9)	60(93.8)		47(78.3)	23(76.7)		43(89.6)	18(78.3)	0.199	
> 13.5	11(9.1)	4(6.2)		13(21.7)	7(23.3)		5(10.4)	5(21.7)		
INR			0.915			0.837			0.635	
< 1.2	111(91.7)	59(92.2)		51(85)	25(83.3)		42(87.5)	21(91.3)		
> 1.2	10(8.3)	5(7.8)		9(15)	5(16.7)		6(12.5)	2(8.7)		
PLT			0.841			0.371			0.128	
< 125	51(42.1)	26(40.6)		28(46.7)	17(56.7)		16(33.3)	12(52.2)		
> 125	70(57.9)	38(59.4)		32(53.3)	13(43.3)		32(66.7)	11(47.8)		
TB			0.422			0.551			0.994	
< 20.4	88(72.7)	50(78.1)		30(50)	17(56.7)		25(52.1)	12(52.2)		
> 20.4	33(27.3)	14(21.9)		30(50)	13(43.3)		23(47.9)	11(47.8)		
Child–Pugh class			0.132			0.273			0.456	
A	89(73.6)	38(59.4)		43(71.7)	23(76.7)		39(81.3)	21(91.3)		
B	22(18.2)	19(29.7)		13(21.7)	3(10)		7(14.6)	2(8.7)		
C	10(8.3)	7(10.9)		4(6.6)	4(13.3)		2(4.1)	0(0)		
AFP			< 0.001			0.004			0.01	0.015
< 20	71(58.7)	22(34.4)		34(56.7)	6(20)		26(54.2)	4(17.4)		
20–400	46(38)	27(42.2)		14(23.3)	11(36.7)		15(33.3)	15(65.2)		
> 400	4(3.3)	15(23.4)		12(20)	13(43.3)		7(14.6)	4(17.4)		
size	33.01 ± 10.4	43.11 ± 11.99	0.002	31.58 ± 18.84	49.30 ± 23.15	< 0.001	28.27 ± 13.56	43.87 ± 14.29	< 0.001	0.03
Radiological Features										
Site			0.286			0.232			0.047	
Left lobe	29(24)	20(31.3)		13(21.7)	10(33.3)		10(20.8)	10(43.5)		
Right lobe	92(76)	44(68.7)		47(78.3)	20(66.7)		38(79.2)	13(56.5)		
Cirrhosis of the liver			0.505			0.436			0.233	
no	34(28.1)	21(32.8)		12(20)	4(13.3)		14(29.2)	10(43.5)		
yes	87(71.9)	43(68.2)		48(80)	26(86.7)		34(70.8)	13(56.5)		
ARE			0.016			0.062			0.156	0.212
no-ring	117(96.7)	55(85.9)		57(95)	24(80)		46(95.8)	19(82.6)		
ring	4(3.3)	9(14.1)		3(5)	6(20)		2(4.2)	4(17.4)		
Radiological capsule			0.208			0.52			0.594	
yes	37(30.6)	14(21.9)		20(33.3)	8(26.7)		11(22.9)	4(17.4)		
no	84(69.4)	50(78.1)		40(66.7)	22(73.3)		37(71.1)	19(82.6)		
tumourmargins			< 0.001			0.004			0.001	0.015
smooth	85(70.2)	18(28.1)		39(65)	10(33.3)		35(72.9)	6(26.1)		
non-smooth	36(29.8)	46(71.9)		21(35)	20(66.7)		13(27.1)	17(73.9)		
internalarteries			0.437			0.111			0.021	
no	95(78.5)	47(73.4)		44(73.3)	17(56.7)		41(85.4)	14(66.7)		
yes	26(21.5)	17(25.8)		16(26.7)	13(43.3)		7(14.6)	9(33.3)		
hypoattenuatinghaloes			0.208			0.255			0.126	
no	114(94.2)	57(89.1)		51(85)	28(93.3)		45(93.8)	18(78.3)		
yes	7(5.8)	7(10.9)		9(15)	2(6.7)		3(6.3)	5(21.7)		
APE			< 0.001			0.048			< 0.001	0.032
no	105(86.8)	37(57.8)		55(91.7)	23(76.7)		43(89.6)	12(52.2)		
yes	16(13.1)	27(42.2)		5(8.3)	7(23.3)		5(10.4)	11(47.8)		

*MVI* microvascular invasion, *HBV* hepatitis B virus, *HCV* hepatitis C virus, *ALT* alanine aminotransferase, *AST* aspartate aminotransaminase, *ALP* alkaline phosphatase, *GGT* glutamyl-transpeptidase, *ALB* albumin, *PT* prothrombin time, *INR* international normalized ratio, *PLT* platelet count, *TB* total bilirubin, *AFP* alpha-fetoprotein, *ARE* arterial rim enhancement, *APE* arterial peritumoral enhancement

^*^multivariate regression analysis

### Development and validation of the nomogram

**Fig. 2:**
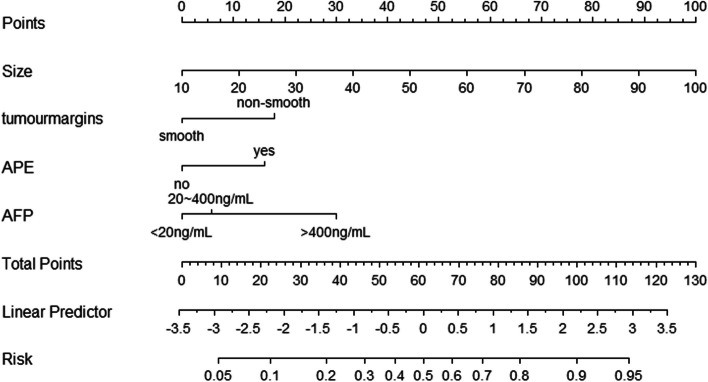
Nomogram of the model. AFP alpha-fetoprotein, APE arterial peritumoral enhancement

**Fig. 3:**
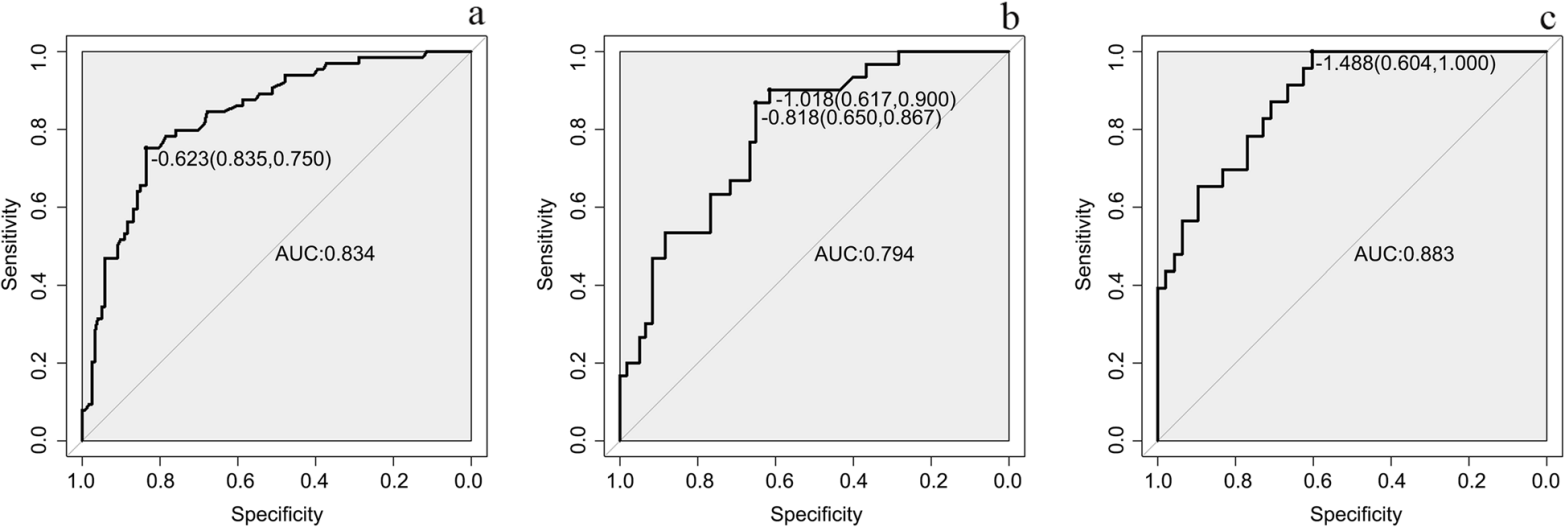
Receiver operating characteristic (ROC) curves of Training, Testing 1 and Testing 2. **a** Receiver operating characteristic (ROC) curves of Training, **b** Receiver operating characteristic (ROC) curves of Testing 1, **c** Receiver operating characteristic (ROC) curves of Testing 2

**Fig. 4:**
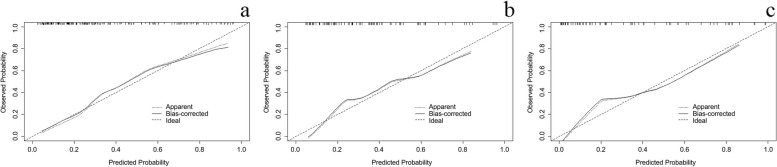
Calibration curves of Training, Testing 1 and Testing 2. **a** calibration curves of training, **b** calibration curves of Testing 1, **c** calibration curves of Testing 2

**Fig. 5:**
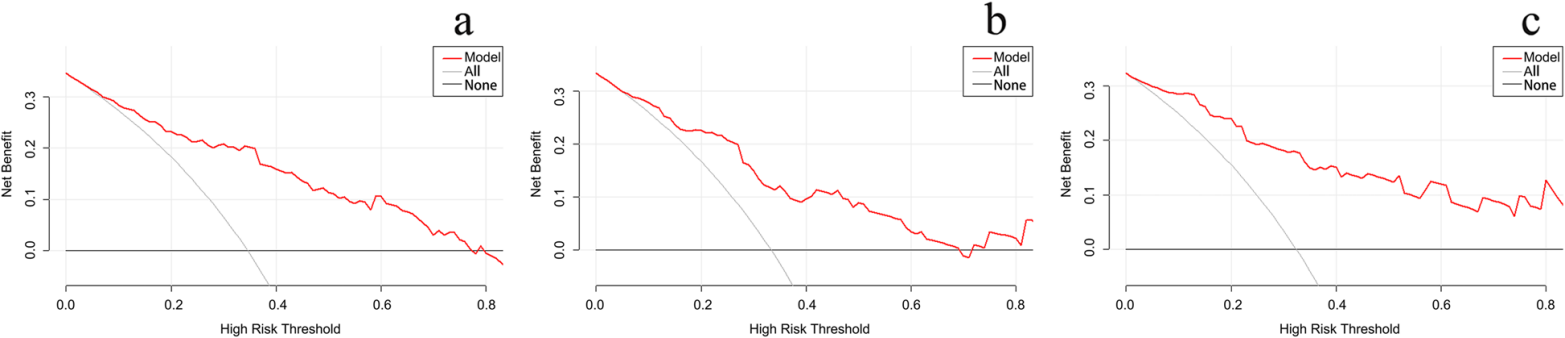
Decision curve of Training, Testing 1 and Testing 2. **a** decision curve of Training, **b** decision curve of Testing 1, **c** decision curve of Testing 2

The nomogram of MVI in HCC is presented in Fig. 2. Among the nomogram predictors, size had the highest score (100 points on the scale axis), followed by AFP, tumour margin and APE (30 points, 18 points and 16 points, respectively). The probability of MVI in HCC can be easily estimated by summing the points of the four variables and locating the corresponding score on the probability axis. The AUCs for the predictive performance of the nomogram were 0.834 (95% CI: 0.774–0.895) in the training dataset, 0.794 (95% CI: 0.700–0.888) in the testing 1 dataset and 0.883 (95% CI: 0.807–0.959) in the testing 2 dataset (see Fig. 3), with no significant difference (*p* > 0.05) (see Table 5). The calibration curves (Fig. 4) showed that in the training (χ^2^ = 5.179, *p* = 0.738), testing 1 (χ^2^ = 6.557, *p* = 0.585) and testing 2 cohorts (χ^2^ = 9.886, *p* = 0.273), the prediction probability of the nomogram was in close agreement with the actual MVI estimate. Nomogram decision curves for the training, test 1 and test 2 datasets are shown in Fig. 5. When the threshold probability is between 0.04 and 0.78 in the training, the predicted net benefits of the nomogram decision curve were higher than those assuming that all patients have MVI. This suggests that our nomogram treatment strategy will improve clinical outcomes.

**Table 5:** The diagnostic performance of nomogram model for MVI in Training, Testing 1 and Testing 2

	AUC	Accuracy	Sensitivity	Specifificity	z	*p*
Training	0.834[0.774,0.895]	80.5	75	83.5	0.701^a^	0.483
Testing 1	0.794[0.700,0.888]	71.1	90	61.7	1.143^b^	0.149
Testing 2	0.883[0.807,0.959]	73.2	100	60.4	0.989^c^	0.322

*MVI* microvascular invasion, *AUC* area under the receiver operating characteristic curve

^a^indicative of AUC comparison between training and testing 1

^b^indicative of AUC comparison between testing 1 and testing 2

^c^indicative of AUC comparison between training and testing 2

## Discussion

Our study demonstrated that a combination of clinical factors, including serum AFP level and tumour size, as well as imaging features, including tumour margin and APE, can predict MVI in HCC. In addition, we established a nomogram that included AFP level, tumour size, tumour margin, and APE to predict MVI with high accuracy and was validated internally and externally. Importantly, the combination of clinical factors and radiological feature in the nomogram affords a direct, noninvasive, and robust method for the personalized prediction in MVI preoperatively.

Of the clinical factors, the results of univariate and multivariate analyses showed that serum AFP level and tumour size were both significant risk factors for MVI, which was consistent with previous reports [21, 26–28]. Serum AFP levels as a marker for HCC have been shown to be associated with factors of tumour aggressiveness including MVI and differentiation [29]. In this study, the serum AFP level was significantly related with MVI, especially when the AFP value was greater than 400 ng/ml, but the sensitivity (67.2%) and specificity (58.7%) were low. The possible reason is that AFP is not a specific marker for HCC, and it can also be elevated to varying degrees in a variety of conditions, such as germ cell tumours, other gastrointestinal tumours, or cirrhosis. According to the Milan criteria, a tumour larger than 5 cm would not be suitable for liver transplantation. However, the critical value of MVI predicted by tumour size varied among studies. Shirabe et al. [30] suggested that a tumour size larger than 3.6 cm was a predictor of MVI. In another study, Kaibori et al. [31] suggested a tumour size larger than 5.0 cm as a predictor of MVI. In our study, we considered a tumour size larger than 3.15 cm to be a predictor of MVI.

Of the imaging features, the results of univariate and multivariate analyses showed that tumour margin and APE were both significant risk factors for MVI. Both tumour margin and APE are features of the peritumoral region. The area around the tumour was the most informative area, including features such as tumour-liver difference, presence or absence of hypodense halo, non-smooth margin, arterial peritumoral enhancement, and arterial rim enhancement. The tumour-liver interface may reflect cellular proliferation, MVI-induced tumour tissue distortion, extracellular matrix remodelling and the associated inflammatory response [32]. An experimental study showed that tumour margins are cross-phonemic points transmitted by the tumour to the host through transforming growth factor-β and platelet-derived growth factor signalling and are therefore critical in tumour cells [33]. In our study, tumour margins were a strong factor in predicting MVI, with an OR value of 2.645.

In this study, we combined the above clinical and imaging features and developed a nomogram. Previous studies using radiomics and deep learning models to predict MVI have achieved a good prediction ability, with AUCs ranging from 0.734 to 0.837 [10, 34]. However, radiomics requires much manual effort, while deep learning also requires many marker samples, and both are poorly interpretable. In clinical application, it is not as convenient and practical as our nomogram model. In addition, we performed external validation. The data in the validation dataset included both MRI and CT data. A published deep learning study revealed that an MRI-based model achieved superior prediction outcomes to a CE-CT-based model [10]. However, our study did not show a significant difference in AUC values between CT and MRI data, which indicated that our nomogram model had high applicability and generalizability and could be used on CE-CT or DCE-MRI data.

Theer are some limitations. First, because of the retrospective nature of this study, there may be potential selection bias. Prospective studies may be needed in the future. Second, the amount of MRI data in the validation dataset was small. Additional data may be required for separate validation in the future. Third, there is no precise evidence of a direct link between radiological features and MVI. Prospective multicentre trials are needed to further investigate the relationship between radiological features and MVI.

In conclusion, serum AFP level, tumour size, tumour margin and APE are potential biomarkers for predicting MVI in HCC patients. Combining clinical factors and imaging features, the nomogram for MVI individualized risk assessment achieves satisfactory preoperative prediction. Moreover, it can be applied to both CT and MRI data with high applicability and generalizability.

## Data Availability

The datasets used and analysed during the current study are available from the corresponding author upon reasonable request.

## Abbreviations

HCC: Hepatocellular Carcinoma
MVI: Microvascular Invasion
CT: Computed Tomography
CE-CT: Contrast Enhancement Computed Tomography
DCE-MRI: Dynamic Contrast Enhancement Magnetic Resonance Imaging
AUC: The area under the receiver operating characteristic curves
T2WI: T2-Weighted Imaging
DWI: Diffusion-Weighted Imaging
T1WI: T1-Weighted Imaging
DTPA: Diethylenetriaminepentaacetic Acid
ROC: Receiver Operating Characteristic curve
HBV: Hepatitis B Virus
HCV: Hepatitis C Virus
ALT: Alanine aminotransferase
AST: Aspartate Aminotransaminase
ALP: Alkaline Phosphatase
GGT: Glutamyl-transpeptidase
ALB: Albumin
PT: Prothrombin Time
INR: International Normalized Ratio
PLT: Platelet count
TB: Total Bilirubin
AFP: Alpha-fetoprotein
ARE: Arterial Rim Enhancement
APE: Arterial Peritumoral Enhancement
SD: Standard Deviation
